# The potential role and mechanism of Rhizoma Coptidis in prevention of diabetic encephalopathy: targeting sodium ion and channels

**DOI:** 10.3389/fphar.2025.1542015

**Published:** 2025-03-14

**Authors:** Ning Cao, Zhangxuan Shou, Mimi Wang, You Wu, Xuefeng Wang

**Affiliations:** ^1^ Pharmacy Department, The Second Affiliated Hospital of Zhejiang Chinese Medical University, Hangzhou, China; ^2^ Department of Neurology, The Second Affiliated Hospital of Zhejiang Chinese Medical University, Hangzhou, China

**Keywords:** Rhizoma Coptidis, diabetic encephalopathy, voltage-gated sodium channels, serum sodium, berberine, coptisine

## Abstract

**Introduction:**

Rhizoma Coptidis (RC) is an edible and medicinal herb with anti-hyperglycemia, which has potential application in the prevention of diabetic encephalopathy (DE). However, its efficacy and underlying mechanism in DE prevention have not been elucidated yet. The objective of the current study is to investigate the preventive effect of RC on DE, thereby focusing on the target through the method of network pharmacology and molecular docking.

**Methods:**

Sixty 4-week-old, male C57BL/6 mice were randomly allocated to six groups: control, model, metformin (200 mg/kg), RCL (0.75 g/kg), RCM (1.5 g/kg), and RCH (3 g/kg). The DE-model mice were induced by streptozocin combined with a high-fat diet. In addition, the neuroprotective effect of RC was determined both *in vivo* and *in vitro*. Network pharmacology analysis was used to screen the potential mechanism of RC. Thereafter, the underlying mechanism of action of RC was explored by molecular docking prediction and Western blot analysis. An analysis of patients with DE was performed to validate it from another perspective.

**Results:**

The results showed that the cognitive state of DE model mice was improved and neuronal injury was ameliorated after RC administration. Active compounds in RC, berberine and coptisine, were found to ameliorate HT22 injury induced by high glucose. Network pharmacology results suggest that voltage-gated sodium channel subtypes (Nav1.1, Nav1.2, and Nav1.6) may be the targets for RC prevention of DE. Furthermore, the Western blot analysis revealed that RC significantly upregulated Nav1.1 and Nav1.2, while Nav1.6 could not. In addition, serum sodium was related to the cognitive status of DE patients, which can be used as a diagnostic index for mild and moderate–severe DE.

**Discussion:**

RC has the potential to be a functional food or adjuvant drug for DE prevention, and Nav1.1 and Nav1.2 are promising DE intervention targets.

## 1 Introduction

Rhizoma Coptidis (RC), called “Huanglian” in Chinese, is an edible herb found in China that has been widely used as a folk treatment due to its role in clearing heat and dampness, purifying fire, and detoxifying the body (Han et al., 2019). In recent years, RC has been used as a food additive and supplemented into some products, such as honey and other functional beverages (Teng and Choi, 2014). With the deepening of research, literature has reported that RC mainly contains isoquinoline alkaloids, lignans, flavonoids, acidic components, etc. (Feng et al., 2020). Modern pharmacological studies have proven that RC has anti-apoptosis, anti-oxidation, nervous system protection, anti-inflammation, anti-tumor, and other effects (Zhou et al., 2023). In addition, with the integration of traditional and modern medicine, RC has been used in assisted treatment of diabetes (Huang et al., 2020).

Diabetic encephalopathy (DE) is a serious neural diabetic complication caused by persistent high blood glucose (Gonz´alez et al., 2020). The encephalopathy associated with diabetes primarily includes working memory, immediate and delayed recall, visual perception, psychomotor speed, executive control, auditory, memory and processing speed, attention, etc. (Chakraborty et al., 2021). Diabetic patients with a diagnosis of less than 12 months have 10% diabetic neuropathy, which increases to as high as 50% at 25 years of age after diagnosis (Liu et al., 2018). Thus, the adoption of appropriate methods to prevent DE after diagnosis of diabetes becomes necessary. RC is a potential drug candidate due to its dual properties of having edible and medicinal values.

Studies have reported that decoctions containing RC, such as Huanglian Jiedu decoction, could inhibit neuronal apoptosis, tau protein hyperphosphorylation, and A*β* deposition (Wang et al., 2020). The active alkaloids of RC, berberine, and coptisine, not only significantly improve the cognitive deficits of model mice and alleviate the nerve damage caused by high glucose but also have the effects of being anti-oxidant, reducing neuroinflammation, and protecting nerve cells (Pereira et al., 2023; Ran et al., 2019; Sadraie et al., 2019). Besides, the reduction of glycated hemoglobin (HbA1c) was found to be significantly associated with RC dose (Huang et al., 2020). *In vivo,* pharmacological experiments demonstrate that RC extract can reverse brain A*β* deposition and neuronal damage and loss (Li et al., 2018). Thus, we can conclude that RC is a promising food and drug candidate for the prevention of DE.

Nevertheless, studies on RC prevention of DE remain incomplete, and its mechanism remains poorly understood. This study aimed to discover and validate the effect and mechanism of RC on DE prevention. In this paper, the pharmacodynamic effects of RC extracts were assessed using DE-model mice induced by streptozocin combined with a high-fat diet. Furthermore, the neuroprotective effect of RC was tested through *in vivo* and *in vitro* studies. Network pharmacology analysis was used to screen the potential mechanism of RC. Subsequently, the mechanism of action of RC was preliminarily explored using molecular docking prediction and Western blot analysis. In addition, an analysis of patients with DE was performed to validate DE from another perspective. Thus, the current study provided a basis for the mechanism of DE prevention and helped to understand the role of RC in a more detailed way.

## 2 Materials and methods

### 2.1 Animals

Sixty 4-week-old, C57BL/6 male mice were obtained from Zhejiang Chinese Medicine University Laboratory Animal Research Center. The animals were housed in a well-lighted air-conditioned room under standard environmental conditions (room temperature 25°C ± 1°C and relative humidity 60%–65%) and given free access to rodent chow and tap water prior to the commencement of the study. All animal-use procedures were in accordance with the regulations for animal experimentation issued and approved by the Animal Ethics Committee of Zhejiang Chinese Medicine University (NO. IACUC-20230327-15; Approval date: 27 March 2023).

### 2.2 RC extracts

The study used an extraction method that was already published previously (Wang et al., 2012). RC (*Coptis chinensis* Franch, specimen number: HL202304) was purchased from Kangmei Pharmaceutical Co., Ltd. RC specimens were smashed to the appropriate granule. Then, 75% ethanol was added to the RC granule for further extraction. The mixture was reflux extracted at 80°C for 1 h. The supernatant of the extract was concentrated to a suitable concentration under reduced pressure. The compounds with the highest content were identified as berberine and coptisine, as illustrated in Supplementary Figure S1.

### 2.3 Drug administration and Morris Water Maze

**FIGURE 1:**
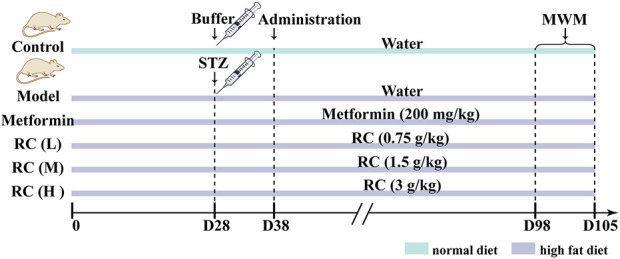
The schedule of drug administration and MWM.

Sixty mice were randomly divided into six groups. One of the groups was fed a control diet and the rest were fed a high-fat diet. After 4 weeks, streptozocin (100 mg/kg) dissolved in sodium citrate-hydrochloric acid buffer solution was intraperitoneally injected into the mice from the group fed with a high-fat diet. The control group received an intraperitoneal injection of sodium citrate-hydrochloric acid buffer. Blood glucose levels were checked 10 days later, and a level higher than 13.4 mM indicated successful induction of diabetes. The mice with successful diabetes induction were randomly divided into five groups: model, metformin (200 mg/kg), RC (L, 0.75 g/kg), RC (M, 1.5 g/kg), and RC (H, 3 g/kg) groups. The dosages of RC were calculated based on the weight of the dried herbal pieces. The control and model groups were orally administered water by gavage, while RC (L, M, H) and metformin groups were administered with corresponding drugs by gavage. The schedule is shown in Figure 1.

The Morris Water Maze (MWM) is a circular water tank surrounded by different visual cues. The tank was whitened by addition of titanium dioxide at 22°C ± 2°C. The tank was separated into four equal quadrants, and a platform was centered in one of the quadrants. The whole experimental procedure was composed of adaptive training (1 day, once a day), visible platform test (1 day, four trials a day), hidden platform tests (5 days, four trials a day), and spatial probe trial (24 h after the last hidden platform test, once a day).

### 2.4 Histopathology assay

Brain tissues were fixed in 4% paraformaldehyde and embedded in paraffin. Then, 5-μm-thick sections were stained with hematoxylin–eosin (HE) to visualize the hippocampus. In addition, the numbers of cells in cornu ammonis 1 (CA1), CA3, and dentate gyrus (DG) subregions of the hippocampus were calculated.

### 2.5 Cell culture and survival rate

Mouse hippocampal neuron cell (HT22) was donated by Prof. Ji Liting from Zhejiang Chinese Medicine University. The cells were cultured in DMEM with 10% FBS and 1% penicillin–streptomycin solution. The cells were cultured in a 5% CO_2_ incubator (temperature 37°C, relative humidity 90%). The inoculation density was 1 × 10^3^ cells/cm^2^.

The cell viability of active alkaloids and glucose was measured by CCK-8 assay. The cells were seeded on a 96-well plate at 1 × 10^4^ cells/mL and cultured in an incubator for 24 h. The medium was replaced with 100 μL of medium containing berberine or coptisine (0.25, 0.5, 1. 2.5, 5, 10, 15, and 30 μM, respectively) or glucose (10, 25, 50, 75, 100, 125, 150, 200 mM, respectively) for 24-h or 48-h incubation. Then, 10 μL of CCK-8 dye was added to each well. After 2 h incubation, the absorbance at 450 nm was measured by a microplate reader (BioTek Instruments, Inc.)

The neuroprotective effect of active alkaloids on HT22 with high glucose injury was determined. First, the cells were incubated with berberine or coptisine at appropriate concentration for 24 h. Then, glucose was added to the cell culture medium for 48-h incubation. Finally, 10 μL of CCK-8 dye was added for detection.

### 2.6 Network pharmacology

All compounds of RC were searched in the Traditional Chinese Medicine Systems pharmacology database and analysis platform (TCMSP, https://old.tcmsp-e.com/tcmsp.php). The potential active compounds in RC were screened with the criteria of oral bioavailability (OB) ≥ 30%, drug-likeness (DL) ≥ 0.18, and blood blood-brain barrier (BBB) ≥ −0.3. Furthermore, we retrieved the targets of active compounds from SwissTarget Prediction database (http://swisstargetprediction.ch/), as well as the TCMSP. The screening condition was limited to “*Homo sapiens*” with a probability filter of >0.1. The targets related to DE pathogenesis were obtained from GeneCards (https://www.genecards.org/) and the OMIM database (https://www.ncbi.nlm.nih.gov/omim/). For SwissTarget Prediction and GeneCards, the top 100 and 500 entries were reserved, respectively.

The two datasets, compound-targets and DE-related-targets, were imported into Venny software. The common targets were input into the String database (https://cn.string-db.org/) to investigate protein–protein interactions (PPI) in the specimen. The organism parameter was set as “*H. sapiens*”, and the interaction confidence score was set as high confidence (0.400).

The potential targets were uploaded to the Metascape online tool (https://metascape.org/) to perform Gene Ontology (GO) enrichment analysis and Kyoto Encyclopedia of Genes and Genomes (KEGG) pathway analysis. GO enrichment was conducted on the aspects of molecular function (MF), biological process (BP), and cellular components (CC). The top 10 GO enrichments and top 20 KEGG pathways were further visualized based on the counts. The statistical significance threshold of enrichment analysis was *P* < 0.05.

### 2.7 Docking and Western blot

According to the result of GO and KEGG enrichment analysis, molecular docking was conducted between five ingredients ((R)-Canadine, berberine, berlambine, coptisine, and palmatine) and three voltage-gated sodium channels (VGSCs, including Nav1.1, Nav1.2, and Nav1.6). The protein structures were acquired from the RSCB Protein Data Bank database (http://www.rcsb.org). The structure of compounds was downloaded as a ligand from the PubChem database. The AutoDock Tool 1.5.6 was employed to perform dehydration and hydrogenation of the proteins. AutoDock Tools 1.5.6 was used to obtain the binding energies in the docking of target proteins with the compounds. The ingredient with the lowest binding energy was selected as the best docking ligand for the formation of the best binding complex. All optimal docking complex patterns were visualized using PyMol 2.1.0 and Proteins Plus (https://proteins.plus).

The expression of VGSCs subunit proteins Nav1.1, Nav1.2, and Nav1.6 was detected through Western blot. The total protein samples were extracted from hippocampus of mice for immunoblotting analysis. Protein concentrations were measured using a BCA kit. The primary anti-Nav1.1 (Cat#PA5-115619, 1:1000, ThermoFisher, Shanghai, China), Nav1.2 (Cat#abs149042, 1:1000, Absin, Shanghai, China) and Nav1.6 (Cat#ab302786, 1:1000, Abcam, Shanghai, China) were used and β-tubulin (Cat#30302ES20, 1:5000, Yeasen, Shanghai, China) was selected as an internal control of total protein.

### 2.8 Electrolyte levels analysis

Data were obtained from the patient files of The Second Affiliated Hospital of Zhejiang Chinese Medical University. The ethics committees of The Second Affiliated Hospital of Zhejiang Chinese Medical University approved the study (Approval No: 2023-LW-033-01). Patients with DE were screened out, and the results of venous blood examination and Mini-Mental State Examination (MMSE) were analyzed. Inclusion criteria were as follows: 1) patients with DE; 2) data available. Exclusion criteria are as follows: 1) electrolyte imbalance; 2) other causes of cognitive impairment. This research focused on the relationship between serum sodium and cognitive status. Patients complying with the above criteria were enrolled in this study, regardless of their age, gender, or medication use. One-way ANOVA was employed to compare the electrolyte levels among patients with cognitive impairment at different stages. Furthermore, the Receiver Operating Characteristic (ROC) curve was calculated.

### 2.9 Statistical analysis

All data were presented as mean ± SD and the statistical analysis was performed using SPSS version 19.0. Statistical differences in data between groups were determined using one-way ANOVA, and followed by a *post hoc* test. In all calculations, statistically significant level was set at *P* < 0.05.

## 3 Results

### 3.1 RC attenuated cognitive dysfunction of DE model mice

**FIGURE 2:**
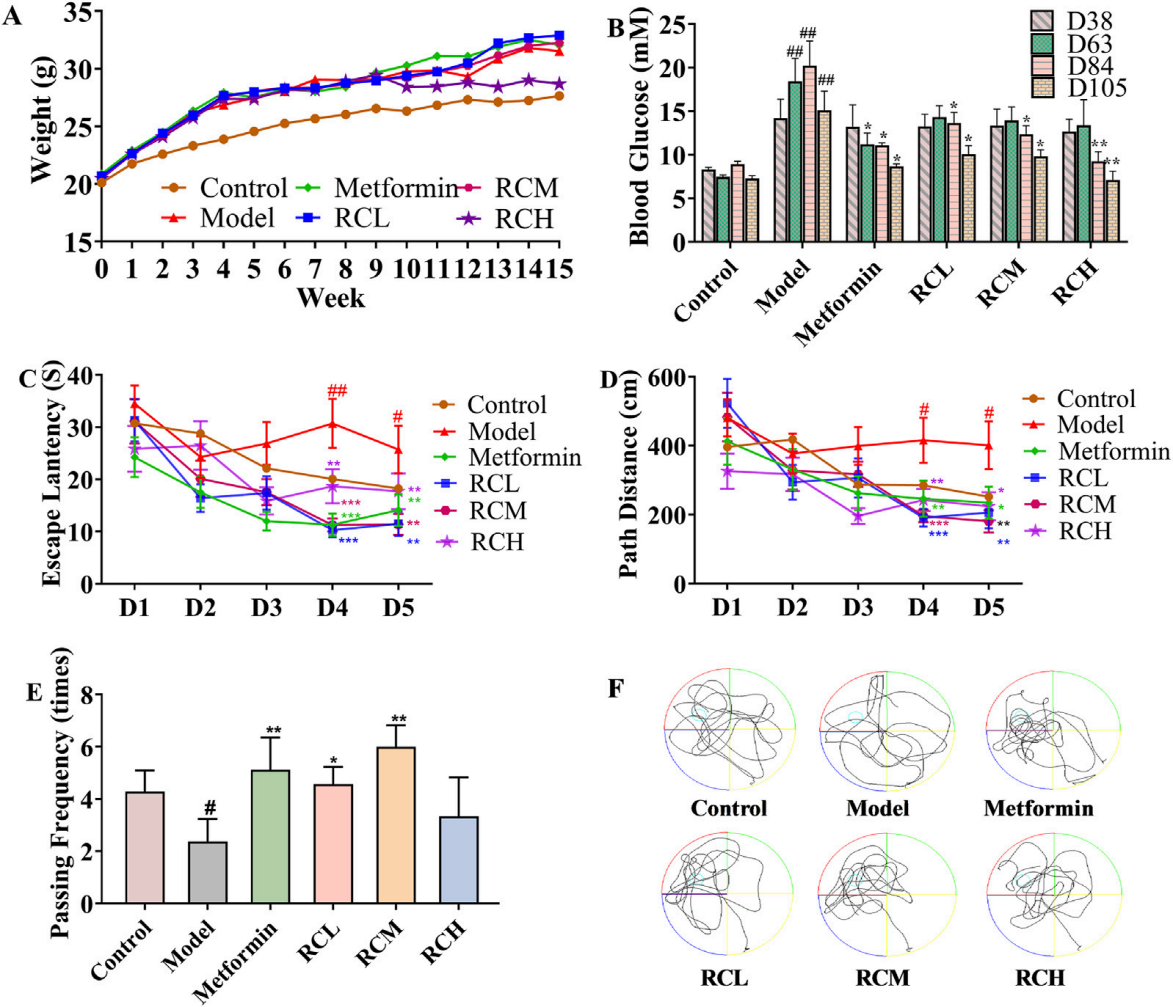
Effects of RC alkaloids on DE. Weight **(A)** and blood glucose **(B)** of each group. Escape latency **(C)**, path distance **(D)**, passing frequency **(E)**, and motion curve **(F)** in MWM test. Data are expressed as mean ± SEM, n = 10, ^#^ *P* < 0.05 and ^##^ *P* < 0.01 vs. control. *P < 0.05, **P < 0.01 and ***P < 0.001 vs. model.

To establish an animal model of DE, a high-fat diet combined with streptozocin was used. As shown in Figure 2A, the weights of mice with a high-fat diet were greater than control. The weight of the mice in each group showed some differences. However, all of the high-fat diet mice did not show any significant difference. In addition, the blood glucose levels of model mice (D63: 18.46 ± 2.62 mM; D84: 20.23 ± 2.82 mM; D105: 15.09 ± 2.21 mM) were significantly higher than that of the control group (D63: 7.46 ± 0.22 mM; D84: 8.92 ± 0.33 mM; D105: 7.27 ± 0.32 mM) throughout the experiment (*P* < 0.01, Figure 2B). In the ninth week, blood glucose levels in the metformin-treated mice (11.23 ± 1.26 mM) were markedly lower than that in the model (18.46 ± 2.62 mM) (*P* < 0.05), while there was no statistically significant change in the RC-treated mice (RCL: 14.30 ± 1.33 mM; RCM: 13.96 ± 1.54 mM; RCH: 13.38 ± 2.94 mM) (*P* > 0.05). Blood glucose levels of RC (L, M, H) treated mice were found significantly lower than the model group at 12th week (RCL: 13.63 ± 1.23 mM; RCM: 12.34 ± 1.00 mM; RCH: 9.25 ± 1.10 mM) and 15th week (RCL: 10.04 ± 1.02 mM; RCM: 9.81 ± 0.76 mM; RCH: 7.12 ± 0.99 mM) (*P* < 0.01). In addition, the average blood glucose levels of the model, metformin, and three RC (L, M, H) treatment groups in the 15th week were lower than those in the 12th week. However, the data did not show any statistically significant differences.

To investigate the effects of RC on spatial learning and memory, the MWM task was carried out in DE model mice. As presented in Figures 2C, D, in the hidden platform tests, the escape latency and path length in model group (fourth day: 30.71 ± 4.71 s, 415.79 ± 65.33 cm; fifth day: 25.73 ± 4.54 s, 401.08 ± 69.21 cm) were significantly longer than those of the control group (fourth day: 20.10 ± 3.23 s, 285.13 ± 47.92 cm; fifth day: 18.24 ± 2.30 s, 252.42 ± 36.54 cm) on the fourth and fifth days (*P* < 0.05). Compared with the model group, the escape latency and path length in mice treated with metformin (fourth day: 11.30 ± 2.15 s, 246.42 ± 52.10 cm; fifth day: 14.12 ± 2.99 s, 234.89 ± 46.42 cm) were significantly reduced on the fourth and fifth day (*P* < 0.05). Meanwhile, RC was found to successfully reverse the cognitive deficits. Compared with model group, the escape latency and path length were effectively reduced in RC (L, M, H) treated groups on the fourth (RCL: 10.29 ± 1.35 s, 191.22 ± 25.53 cm; RCM: 11.25 ± 1.22 s, 196.29 ± 18.28 cm; RCH: 18.69 ± 3.29 s, 242.93 ± 32.30 cm) and fifth day (RCL: 11.50 ± 2.35 s, 206.19 ± 45.60 cm; RCM: 11.38 ± 2.00 s, 180.88 ± 32.32 cm; RCH: 17.71 ± 3.43 s, 224.09 ± 40.82 cm) (*P* < 0.05).

On the day after the hidden platform test, the spatial probe trial was conducted by taking the platform away to evaluate the spatial memory of all mice. As shown in Figures 2E, F, the passing frequency showed a significant decline in the model group (2.38 ± 0.86 times) compared with the control (4.28 ± 0.81 times) (*P* < 0.05). The passing rates of mice in the RC treatment groups (RCL:4.57 ± 0.65 times; RCM: 6.00 ± 0.82 times) and the metformin group (5.13 ± 1.23 times) were found significantly higher than those in the model group (*P* < 0.05).

### 3.2 Neuroprotective effects of RC on the hippocampus

**FIGURE 3:**
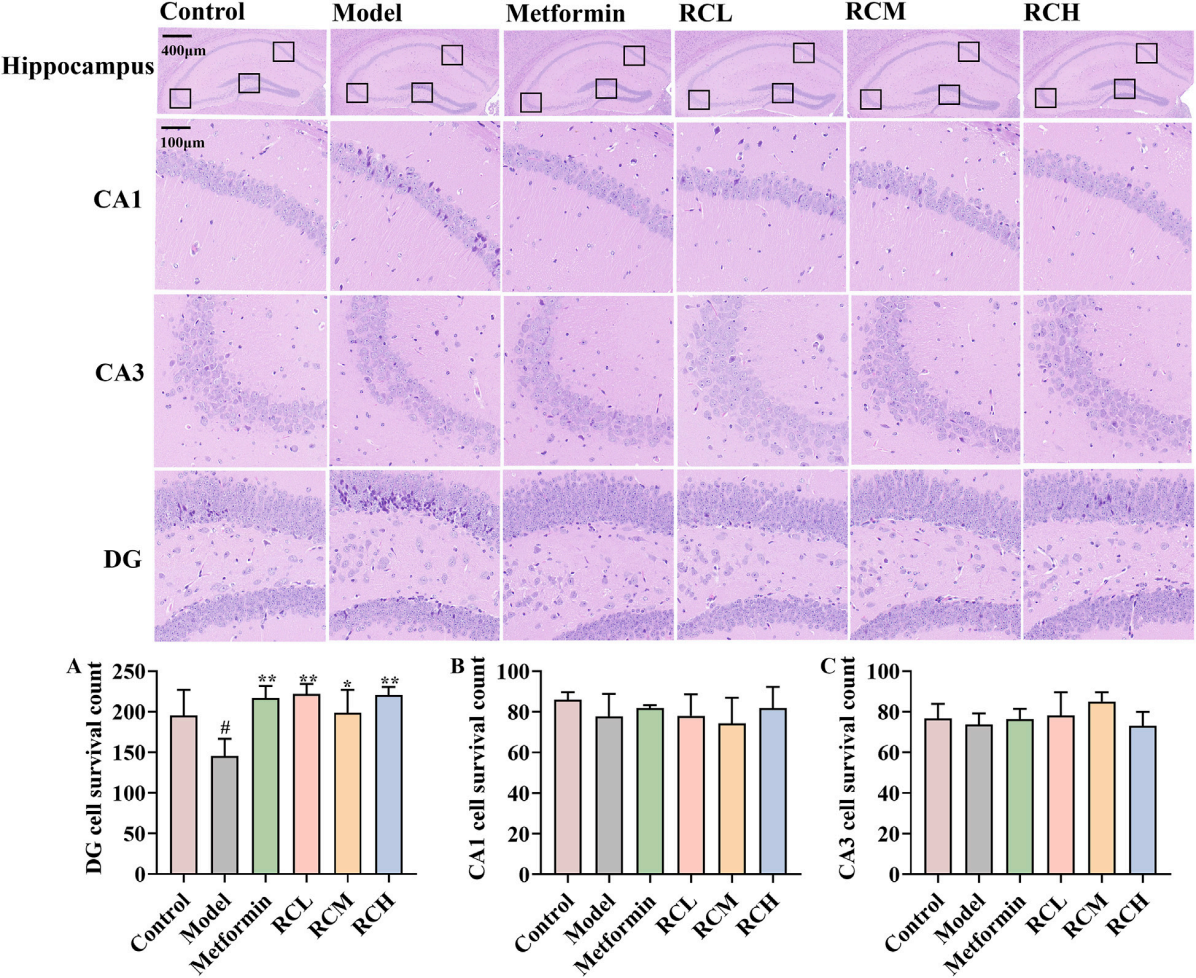
Effects of RC alkaloids on histopathology of brain in DE mice. The effect of RC alkaloids on the DG **(A)**, CA1 **(B)** and CA3 **(C)** pyramidal cells survival count. Data are expressed as mean ± SD, n = 3, ^#^ *P* < 0.05 vs. control. *P < 0.05 and **P < 0.01 vs. model.

The hippocampus plays an important role in learning and memory. As the results of the HE staining assay showed in Figure 3, compared with the control group (195.33 ± 31.66), model mice (145.00 ± 21.52) displayed pathological inflammatory features including reduced cellular layers (Figure 3A, *P* < 0.05), hyperchromatic neurons with increased intercellular spaces in hippocampal DG. Moreover, RC extracts (RCL: 221.67 ± 12.58; RCM: 198.67 ± 28.38; RCH: 220.33 ± 10.02) could alleviate the pathological changes and protect the hippocampal cells of DE model mice (*P* < 0.05).

**FIGURE 4:**
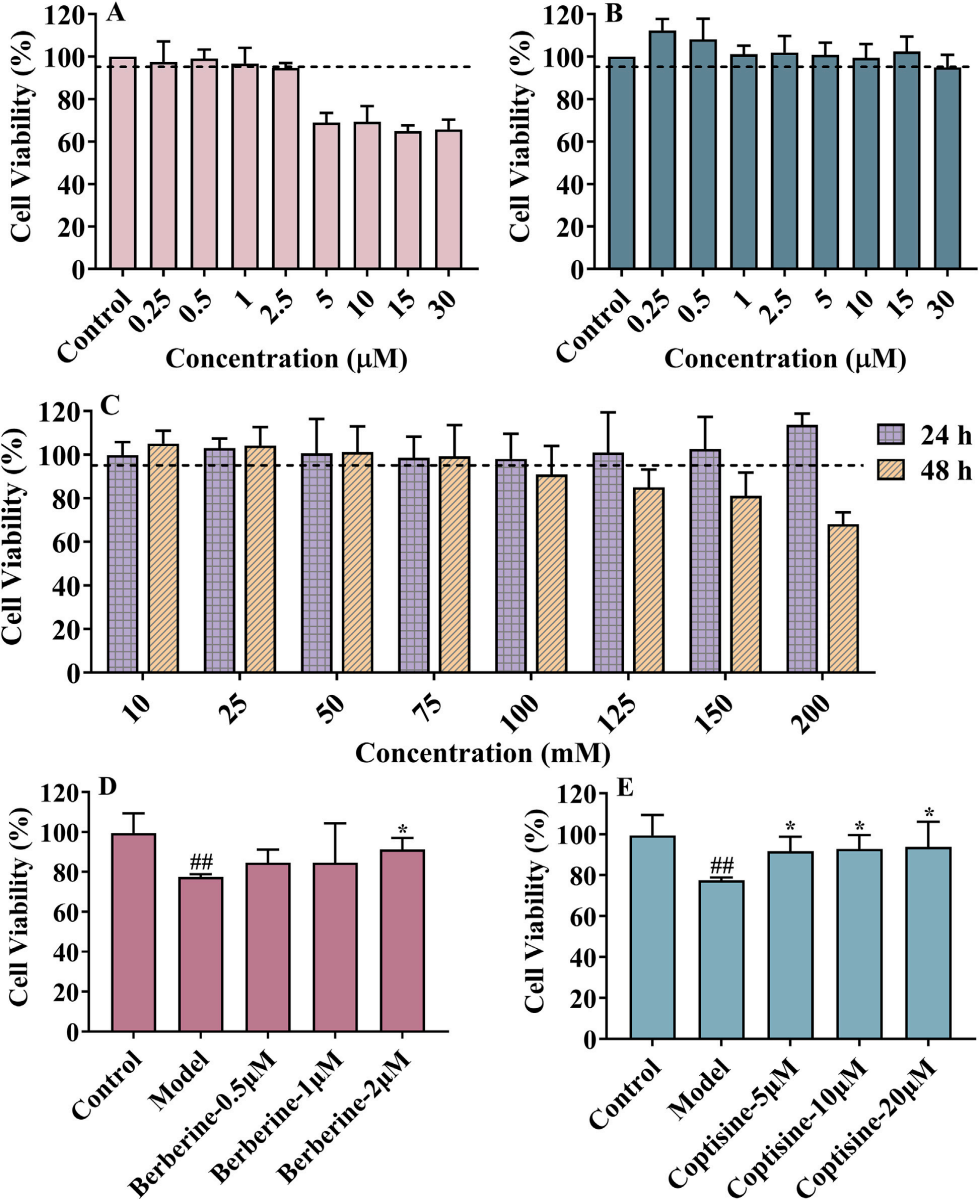
The cytotoxic effects of berberine **(A)**, coptisine **(B)** and glucose **(C)**; Neuroprotective effects of berberine **(D)** and coptisine **(E)** on HT22 injury induced by high glucose levels. Data are expressed as mean ± SD, n = 6, ^#^ *P* < 0.05 vs. control. *P < 0.05 vs. model.

Due to the HPLC result of RC extract, berberine and coptisine, were tested with CCK-8 assay for possible cytotoxic effects in the HT22 cell. Furthermore, 5 μM berberine and 30 μM coptisine demonstrated no significant cytotoxic effect according to the survival rate (Figures 4A, B). In addition, the cell viability was less than 95% when cells were incubated with 100 mM glucose for 48 h (Figure 4C). In subsequent experiments, HT22 was treated with 150 mM glucose for 48 h as a model group. As shown in Figures 4D, and E, the survival rate of model group cells was significantly lower than that of the control group (*P* < 0.05). Meanwhile, the survival rate of HT22 cells treated with berberine (2 μM) and coptisine (2, 5, 10 μM) was significantly higher than the model group (*P* < 0.05, Figures 4D, E).

### 3.3 Network pharmacological analysis for RC

**FIGURE 5:**
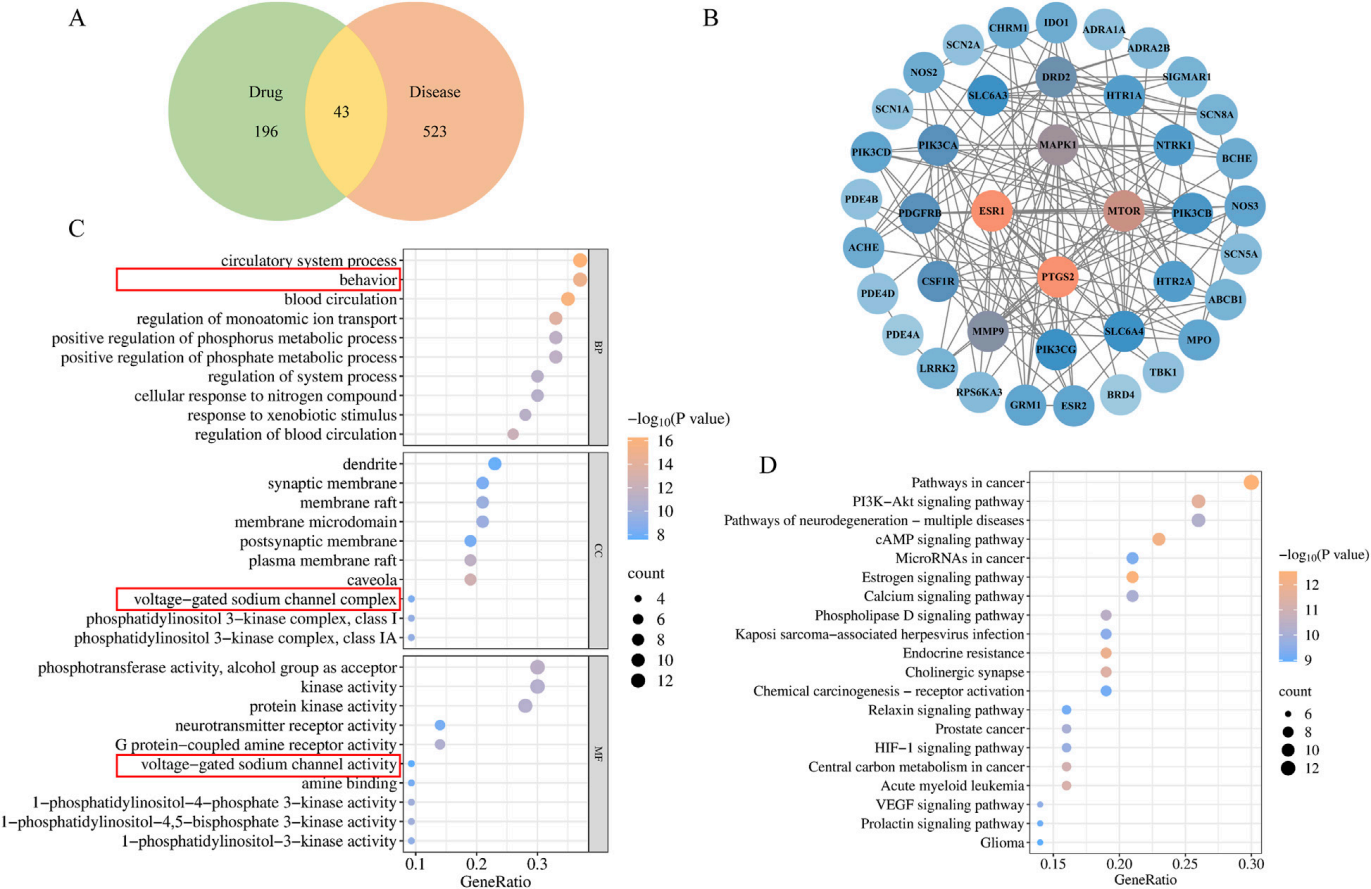
Network pharmacology of RC in DE treatment. **(A)** Venn diagrams illustrated overlapping targets, **(B)** PPI network of RC and DE intersection targets; GO **(C)** and KEGG **(D)** analysis of intersection targets.

In total, 9 ingredients and 239 active ingredient targets of RC were acquired through TCMSP and SwissTarget Prediction database. A total of 566 targets related to DE were obtained in the GeneCards and OMIM. As a result, Venn diagrams illustrated 43 overlapping targets were identified, suggesting these as potential targets for RC in DE treatment (Figure 5A). In addition, a PPI network of potential targets was analyzed using Cytoscape software to examine the interactions among the 43 screened targets (Figure 5B). Details of the compounds corresponding to these 43 targets are shown in Supplementary Table S1.

To investigate the complex mechanism of RC activity against DE, enrichment analyses of the GO and KEGG analyses were performed. In addition, 616 related pathways were significantly obtained with standard *P* < 0.01. GO enrichment analysis results involving blood circulation, behavior, regulation of monoatomic ion transport, voltage-gated sodium channel activity, voltage-gated sodium channel complex, etc. (Figure 5C). Additionally, the top 20 enriched KEGG signaling pathways were demonstrated in Figure 5D, including pathways of neurodegeneration: multiple diseases, and endocrine resistance. Based on the aforementioned results, genes SCN1A, SCN2A, SCN5A, and SCN8A, were screened. According to the National Center for Biotechnology Information (NCBI), Nav1.5, encoded by SCN5A, is found primarily in cardiac muscle, not the brain. Therefore, Nav1.1, Nav1.2, and Nav1.6, encoded by SCN1A, SCN2A, and SCN8A respectively, were verificated. Compounds targeting Nav1.1, Nav1.2, and Nav1.6 include (R)-Canadine, berberine, berlambine, coptisine, and palmatine, as shown in Supplementary Table S1.

### 3.4 Potential mechanism of RC to prevent DE

**FIGURE 6:**
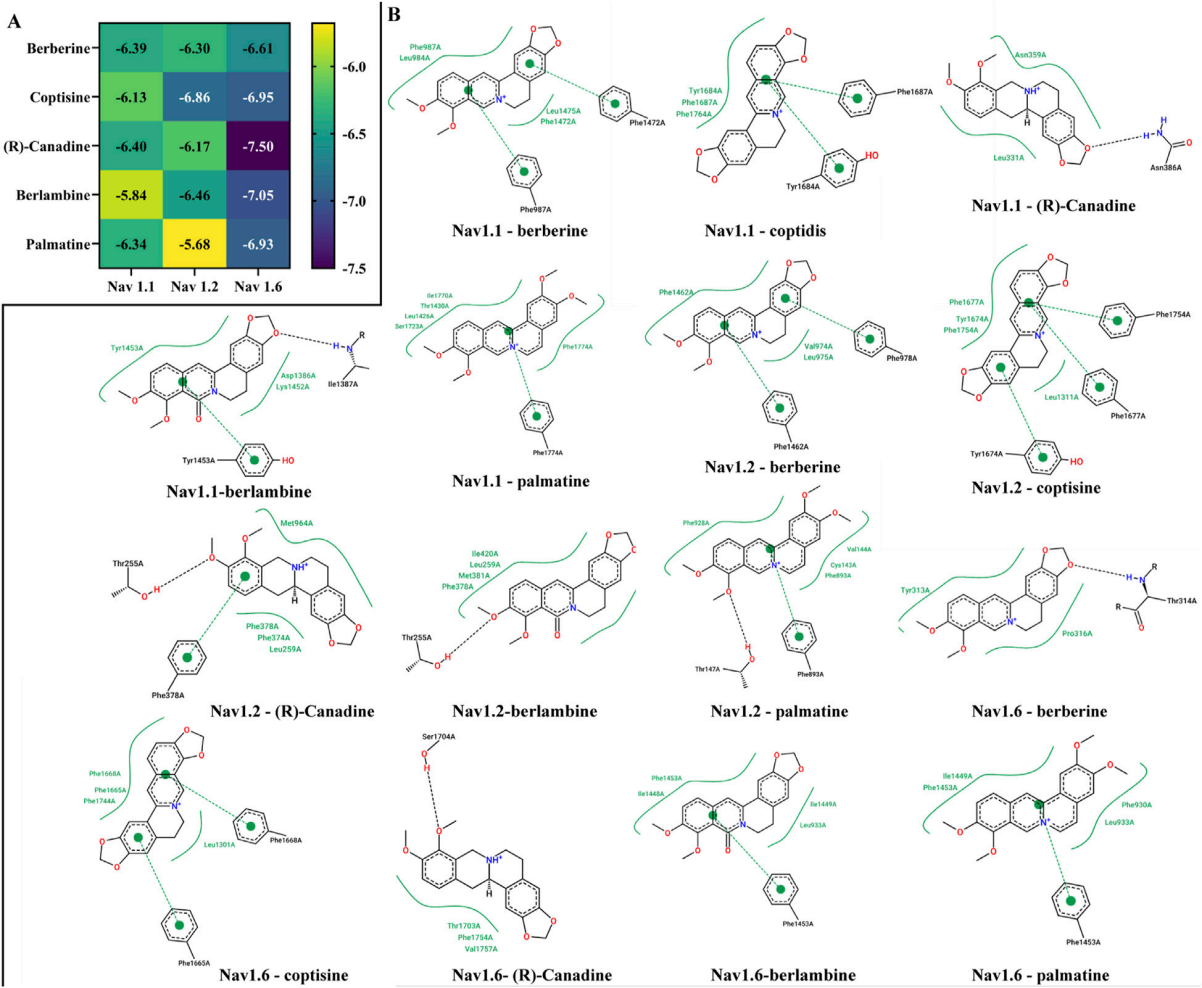
Binding energy **(A)** and binding site of key bioactive ingredients of RC extract with Nav 1.6, Nav1.1, Nav1.2 **(B)**.

Docking active compounds with the ion channels helps verify the results of GO and KEGG enrichment. Subsequently, semi-flexible molecular docking simulations were conducted between these corresponding ingredients ((R)-Canadine, berberine, berlambine, coptisine, and palmatine) and the potential key targets VGSCs (Nav1.1, Nav1.2, and Nav1.6). The outcomes of the binding energy are presented in Figure 6A. The results revealed that all targets had a score below −5.68 kcal/mol. In general, the binding energy of Nav1.6 with the compounds was lower than that of Nav1.1 and Nav1.2. In addition, Figure 6B portrays the visual representation of the receptor and ligand complex with the lowest binding energy. The 2D interaction diagrams showed some differences in the interactions between the compound and the targets, indicating the need to further explore bioactive center in future studies.

**FIGURE 7:**
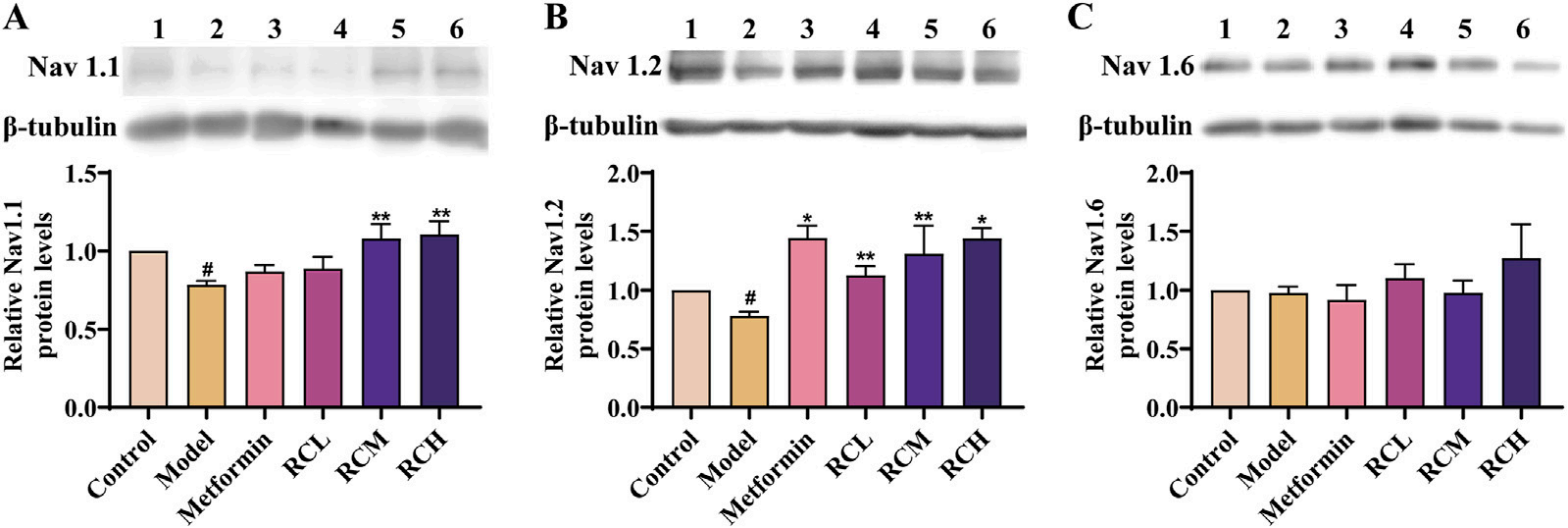
Expression of Nav 1.1 **(A)**, Nav 1.2 **(B)** and Nav 1.6 **(C)** in each group of mice.

The expression levels of Nav1.1, Nav1.2, and Nav1.6 were examined to further validate the results obtained from network pharmacology. As shown in Figure 7, there were significant differences in the expression levels of Nav1.1 and Nav1.2 between the control and model mice (*P* < 0.05), while no significant difference was observed in the expression of Nav1.6. In addition, metformin and RC extracts (L, M, H) significantly upregulated the expression levels of Nav1.2 in the model mice. In the groups treated with RCL and metformin, there was no significant change in the expression levels of Nav1.1, whereas in the RCM and RCH treatment groups, the expression levels of Nav1.1 were upregulated (*P* < 0.01).

### 3.5 Serum sodium levels in DE patients with different cognitive status

**FIGURE 8:**
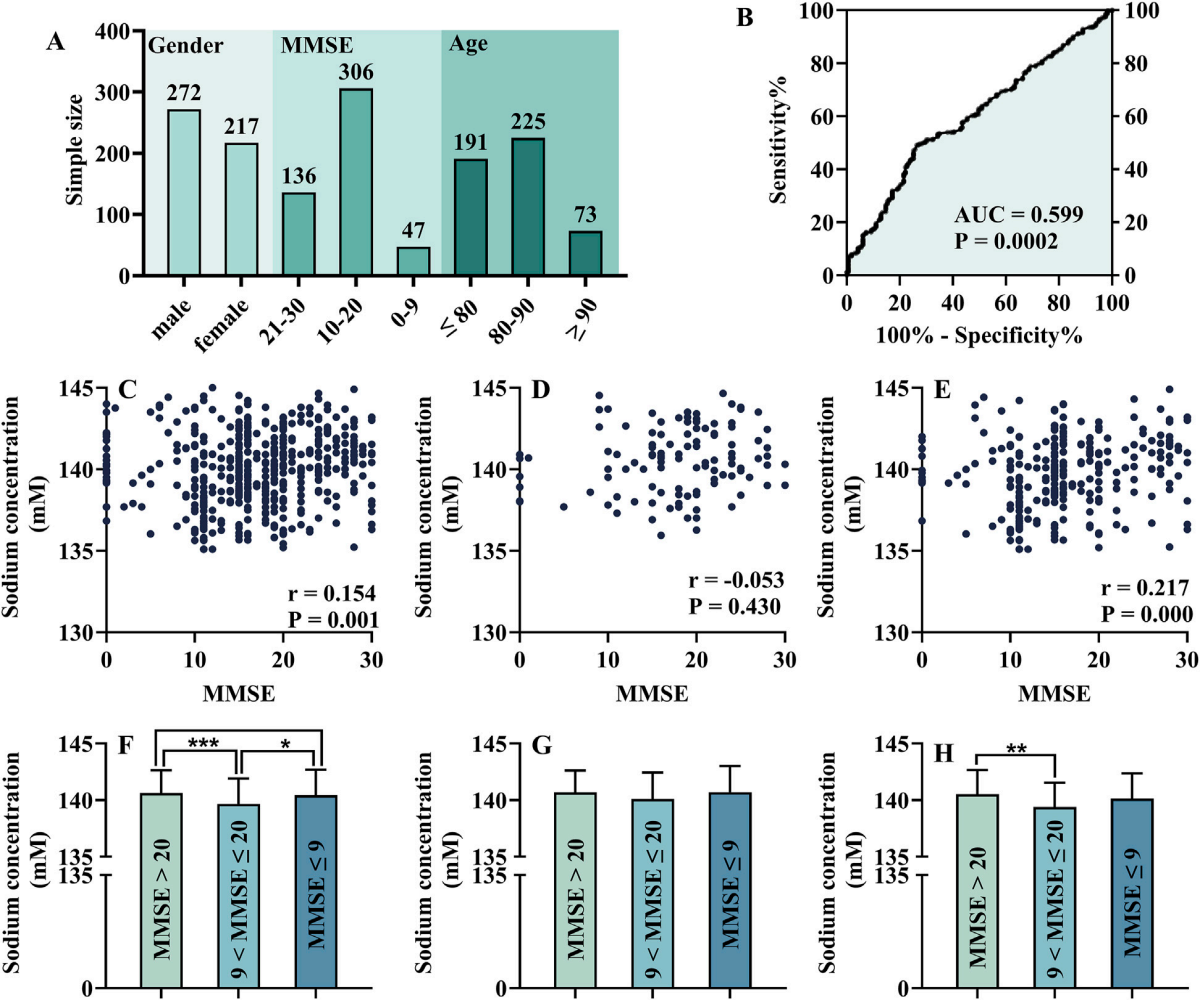
Analysis of each electrolyte level in different cognitive status: general information of participants **(A)**, ROC test of serum sodium **(B)**, Spearman correlation analysis of MMSE score and serum sodium concentration [**(C)**, including female **(D)** and male **(E)**], One-way ANOVA of the electrolyte levels in patients with cognitive impairment at different stages [**(F)**, including female **(G)** and male **(H)**].

Electrolyte levels play an essential role in metabolic disease. As shown in Figure 8A, there were 489 participants in this study. Among them, the male-to-female ratio was 272:217, the ratio of subjects with mild, moderate, and severe cognitive impairment was 136:306:47. The majority of participants were between 80 and 90 years old. According to Spearman correlation analysis, the MMSE score was positively related to sodium concentration (r = 0.154, *P* = 0.001, Figure 8C). In addition, there was a correlation between MMSE score and sodium concentration in male participants (r = 0.216, *P* = 0.000, Figure 8E), but not in female participants (Figure 8D). Then, all participants were divided into three groups according to different stages of DE. As shown in Figure 8F, the concentration of sodium showed a trend of first decreasing and then increasing with the progression of DE disease, which may explain the low correlation coefficient. However, female (Figure 8G) and male (Figure 8H) participants were different in this trend. Furthermore, the ROC curve showed that sodium concentration could be used as a diagnostic index for mild and moderate–severe DE (AUC = 0.599, *P* = 0.0002, Figure 8B). However, DE participants often require long-term medication, which is an inevitable interference factor in this study.

## 4 Discussion

The aforementioned studies indicate that RC exerts a certain effect on DE prevention, with Nav1.1 and Nav1.2 identified as potential targets. Meanwhile, there are some limitations in this study. First, a small sample size and DE modeling method may lead to potential bias. In addition, the potential clinical relevance of serum sodium is limited by drugs, diseases, and other factors. Further research strictly distinguished the need for various clinical conditions. In addition, molecular docking is the prediction of VGSCs binding to RC active alkaloids. Specific biochemistry experiments are needed to confirm this prediction. Finally, it's worth noting that only apparent changes in VGSC protein content have been observed, while their specific role in DE has not been fully and unequivocally demonstrated. Based on their ion channel properties, further validation of the functions of Nav1.1 and Nav1.2 is necessary, particularly in nerve conduction, such as dynamic characteristics, and voltage-dependent fast inactivation.

According to Chinese pharmacopeia and clinical research, the clinical conventional dose of RC is approximately 5–10 g per adult in China (the equivalent surface area dose of the mouse is approximately 0.75–1.50 g/kg) (Ma and Ma, 2013). Meanwhile, the median lethal dose (LD_50_) of the oral RC in mice was 4.9 g/kg, thereby indicating a narrow therapeutic window (Ma and Ma, 2013). Based on the aforementioned MWM results, mice in the RCL and RCM groups showed significant improvement in the spatial probe test, whereas no significant improvement was observed in the RCH group. This may suggest potential damage in mice at high doses of RC. Clinically, RC has been associated with respiratory failure, extrapyramidal reactions, severe arrhythmias, liver function damage, and so on (Wang J. et al., 2019). The neurological damage caused by high-dose RC remains to be investigated. When applying this finding to humans, further research may be necessary to determine the specific clinical dose of RC.

RC and its bioactive alkaloids have the potential to suppress diabetes-induced lesions, including vascular dysfunction, encephalopathy, and retinopathy (Ran et al., 2019). Berberine has demonstrated therapeutic effects on neurological diseases, including Alzheimer’s disease, depressive disorder, and so on (Song et al., 2020). Besides, coptisine could decrease the activation of microglia and astrocytes, reduce amyloid plaque formation, and ameliorate impaired cognition of APP/PSⅠ mice (Yu et al., 2015). The effect of RC on DE has been reported while the effective substances are not clearly understood. Many studies pay attention to berberine but other compounds are ignored, which results in a one-sided view. Studies focusing on the synergistic effect of different compounds are necessary.

Establishing a stable DE modeling method is crucial for an experiment. The DE model mice and cells are derived from type 2 diabetes, which is a debatable method to some extent. However, most scholars adopt the modeling method of this study, due to its characteristics of diabetes complications (Verma and Despa, 2019). According to the preliminary experiment, the higher the blood glucose, the better is the modeling rate of mice. In addition, most DE model mice are more common in male than female rats, while DE shows some sex-dimorphic features. Sex steroids are synthesized in the peripheral gland as well as directly in the nervous system and are important physiological regulators of nervous function (Falvo et al., 2023). Sex steroids may be a potential risk of DE, and research focus on female DE mice is necessary.

The effect of RC extract on DE may be a combination of hypoglycemic and cognitive improvement. Blood glucose was monitored throughout the experiment and decreased during this period in each group except for model and control mice. The blood glucose levels of each group of mice on D108 were lower than those on D84. The MWM may explain the fluctuations in blood glucose. American College of Sports Medicine and the American Diabetes Association have published a consensus statement that type 2 diabetes patients should engage in physical activity regularly and be encouraged to reduce sedentary time (Kanaley et al., 2022). Strength training was reported to be superior to aerobic training alone for reducing HbA_1c_ in patients with type 2 diabetes (Kobayashi et al., 2023). A randomized controlled trial of diabetic peripheral neuropathy showed that 6 months of structured strength and balance training resulted in sustained improvement in functional status (Venkataraman et al., 2019). In addition, physical activity tai chi chuan was found effective in improving global cognitive function in older DE patients, especially in long-term benefit (Chen et al., 2023). Further studies are needed to investigate the ameliorating effects of swimming on DE and diabetes.

There is a need to further explore the underlying mechanism of RC in DE-prevention based on VGSCs. VGSC subtypes, including Nav1.1, Nav1.2, and Nav1.6, play an essential role in various cognitive disorders such as Alzheimer’s disease, and chronic cerebral hypoperfusion (Hu et al., 2019; Martinez-Losa et al., 2018; Yuan et al., 2022). In addition, berberine affects the electrophysiological state of the DG and CA1 region of the hippocampus in DE model rats, including excitatory postsynaptic potential and group peak potential, and improves synaptic plasticity (Ran et al., 2019). In addition, berberine has been found to inhibit autophagy by inhibiting the c-JNK signaling pathway, while coptisine enhances the production of reactive oxygen species and activates the JNK signaling pathway (Ran et al., 2019). Furthermore, JNK is an important regulator of VGSCs, and inhibition of the JNK signaling pathway reduces the APP-induced increase of sodium current and the expression of some VGSCs subtypes (Li et al., 2016). Therefore, the function of VGSCs in preventing DE may be related to the JNK signaling pathway, which requires further investigation.

VGSCs play an indispensable role in neurotransmitter homeostasis; meanwhile, abnormalities in neurotransmitter levels in the brain are considered hallmarks of abnormal brain conditions (Sarter et al., 2006). Various studies have reported the relationship between VGSCs and neurotransmitters. Stimulation of VGSCs could prolong the depolarization of the nerve terminal, thereby promoting neurotransmitter release (Collaço et al., 2019). Loss-of-function mutation of Nav1.1 in model mice showed diminished gamma-aminobutyric acid-ergic (GABAergic) interneuron function, with an inability to sustain repetitive firing in GABAergic interneurons of the cortex and hippocampus (Martin et al., 2010). In addition, dopaminergic cell loss could be prevented efficiently by VGSC agonists (Salthun-Lassalle et al., 2004). The expression of Nav1.1 was remarkably increased 4 weeks after injection of 6-hydroxydopamin, while the expression of Nav1.6 was not significantly different (Wang Z. et al., 2019). However, 49 days after 6-hydroxydopamin injection, the expression of Nav1.1 was sharply reduced, and the expression of Nav1.6 was increased (Wang Z. et al., 2019). Fluctuations in neurotransmitter levels may help explain the function of VGSCs in DE.

Nav1.1 and Nav1.2 are promising targets for the treatment of DE due to the different expressions in DE model mice compared to the control. VGSCs are essential ion channels for nerve cells. The expression of Nav1.2 is increased in the hippocampus and cortex of chronic cerebral hypoperfusion model rat, while repression of the increased expression of Nav1.2 improves neuropathological and cognitive impairment (Hu et al., 2019). In human amyloid precursor protein transgenic mice, increased Nav1.1 levels accelerated action potential kinetics of fast-spiking and non-fast-spiking neurons and improved cognitive function (Martinez-Losa et al., 2018). There are several studies on VGSCs and other cognitive disorders, including Alzheimer’s disease, vascular dementia, and so on, whereas there are no studies on DE (Hu et al., 2019; Kim et al., 2011; Martinez-Losa et al., 2018). Nav1.1 and Nav1.2 are mainly expressed in the brain, and Nav1.6 is expressed in central and peripheral nervous systems according to NCBI data. Thus, the regulation of RC extract on Nav1.1 and Nav1.2 may indicate targeting therapy of central nervous diseases, and fewer side effects on the peripheral nervous system.

In addition to affecting neurotransmitters, VGSCs are essential for sodium balance. Serum sodium balance is considered related to cognitive state, but the research on DE has not yet been reported. According to the research of the British Geriatrics Society, low serum sodium (≤137 mmol/L) was found associated with worse cognitive assessments (van der Burgh et al., 2023). In addition, lower serum sodium was associated with a higher risk of cognitive health in middle-aged and elderly people, but not with the incidence of dementia (van der Burgh et al., 2023). Furthermore, a cohort study of osteoporotic fractures in men showed that serum sodium within a certain range was associated with cognitive decline (Nowak et al., 2018). Thus, the serum sodium concentrations reflect cognitive condition to some extent, which is consistent with the present study. However, there are some limitations in this analysis. MMSE used in this study is influenced by race, ethnicity, education level, and socioeconomic status, which often requires different scoring criteria for certain individuals (Jannati et al., 2024). In addition, serum sodium is influenced by various elements, including salt intake, habits, and medication (Kuwabara et al., 2020). Finally, aging is a significant risk factor for cognitive dysfunction (Du Preez et al., 2024). In this study, most participants were aged over 80 years, which makes it somewhat difficult to disentangle cognitive impairments caused by DE and aging. Detail randomized controlled trials involving younger participants may help to clarify these ambiguities.

## 5 Conclusion

In this study, we found that RC does prevent the progression of DE in model mice, protecting hippocampal neurons. Nav1.1 and Nav1.2 are potential targets in the prevention of DE. According to the analysis of patients with DE, serum sodium, as an essential substrate of VGSCs, can be a potential diagnostic index for mild and moderate-severe DE. This study provides an experimental basis for the daily prevention and clinical application of RC and the precautionary mechanism of DE. Overall, the synergistic effect of RC’s different active components on VGSCs and their relative pathway is a promising direction for future research.

## Data Availability

The raw data supporting the conclusions of this article will be made available by the authors, without undue reservation.

## Abbreviations

BP, biological process; CA1, cornu ammonis 1; CC, cellular components; DE, diabetic encephalopathy; DG, dentate gyrus; DL, drug-likeness; GABAergic, gamma-aminobutyric acid-ergic; GO, Gene ontology, HbA1c, glycated hemoglobin; JNK, c-Jun N-terminal kinase; KEGG, Kyoto encyclopedia of genes and genomes; MF, molecular function; MWM, Morris Water Maze; MMSE, Mini-Mental State Examination; OB, oral bioavailability; PPI, protein-protein interactions; RC, Rhizoma Coptidis; ROC, Receiver Operating Characteristic; TCMSP, Traditional Chinese Medicine Systems pharmacology database and analysis platform; VGSCs, voltage-gated sodium channels.
