# Supplementary material for: The potential role and mechanism of Rhizoma Coptidis in prevention of diabetic encephalopathy: targeting sodium ion and channels
Source: Front Pharmacol. 2025 Mar 14;16:1542015. doi: 10.3389/fphar.2025.1542015 (PMC11949989; doi:10.3389/fphar.2025.1542015)
Supplement: Supplementary file 1 [file DataSheet1.docx]

Figure S1: Chromatogram of RC extraction indicating the identified major compounds, [1] berberine, [2] coptisine. An Agilent 1260 Infinity HPLC system was used for analysis. The chromatographic column was Kromasil C18 Column(250 mm × 4.6 mm, 5 μm). 0.1% formic acid solution was used as mobile phase A, while methanol served as mobile phase B. The column temperature was set at 30 °C, and the flow rate was maintained at 0.8 mL/min. Gradient elution was employed during the experiment: 0-8min, 10%-25%B; 8min-14min, 25%-30%B; 14min-18min, 30%B; 18min-26min, 30%-60%B; 26min-35min, 60%-71%B; 35min-43min, 71%-90%B; 43min-55min, 90%B.

Table S1 Compounds corresponding to the targets

| Compound | Target |
| --- | --- |
| (R)-Canadine | ADRA1A |
| (R)-Canadine | CHRM1 |
| (R)-Canadine | CHRM5 |
| (R)-Canadine | DRD2 |
| (R)-Canadine | HTR1A |
| (R)-Canadine | HTR2A |
| (R)-Canadine | PTGS2 |
| (R)-Canadine | SCN5A |
| (R)-Canadine | SIGMAR1 |
| (R)-Canadine | SLC6A3 |
| (R)-Canadine | SLC6A4 |
| Berberine | ACHE |
| Berberine | ADRA2B |
| Berberine | BCHE |
| Berberine | CHRM1 |
| Berberine | ESR1 |
| Berberine | NOS2 |
| Berberine | NOS3 |
| Berberine | PTGS2 |
| Berberine | SCN1A |
| Berberine | SCN2A |
| Berberine | SCN5A |
| Berberine | SIGMAR1 |
| Berberrubine | ACHE |
| Berberrubine | ADRA2B |
| Berberrubine | BCHE |
| Berberrubine | CHRM1 |
| Berberrubine | ESR1 |
| Berberrubine | NOS2 |
| Berberrubine | NOS3 |
| Berberrubine | PTGS2 |
| Berberrubine | SCN5A |
| Berberrubine | SIGMAR1 |
| Berlambine | ABCB1 |
| Berlambine | ACHE |
| Berlambine | ADRA2B |
| Berlambine | BCHE |
| Berlambine | CHRM1 |
| Berlambine | DRD2 |
| Berlambine | GRM1 |
| Berlambine | IDO1 |
| Berlambine | MAPK1 |
| Berlambine | NOS2 |
| Berlambine | NOS3 |
| Berlambine | PDE4A |
| Berlambine | PDE4B |
| Berlambine | PDE4D |
| Berlambine | PIK3CA |
| Berlambine | PIK3CB |
| Berlambine | PTGS2 |
| Berlambine | RPS6KA3 |
| Berlambine | SCN5A |
| Berlambine | SCN8A |
| Berlambine | SIGMAR1 |
| Berlambine | TBK1 |
| Coptidis | SCN1A |
| Coptidis | SCN2A |
| Coptidis | ACHE |
| Coptidis | ADRA2B |
| Coptidis | BCHE |
| Coptidis | CHRM1 |
| Coptidis | ESR1 |
| Coptidis | NOS2 |
| Coptidis | NOS3 |
| Coptidis | PTGS2 |
| Coptidis | SCN5A |
| Coptidis | SCN8A |
| Coptidis | SIGMAR1 |
| Epiberberine | ACHE |
| Epiberberine | ADRA2B |
| Epiberberine | BCHE |
| Epiberberine | CHRM1 |
| Epiberberine | ESR1 |
| Epiberberine | NOS2 |
| Epiberberine | NOS3 |
| Epiberberine | PTGS2 |
| Epiberberine | SIGMAR1 |
| Magnograndiolide | ABCB1 |
| Magnograndiolide | BRD4 |
| Magnograndiolide | DRD2 |
| Magnograndiolide | LRRK2 |
| Magnograndiolide | MPO |
| Magnograndiolide | NOS2 |
| Magnograndiolide | PREP |
| Magnograndiolide | PTGS2 |
| Palmatine | ACHE |
| Palmatine | ADRA2B |
| Palmatine | BCHE |
| Palmatine | CHRM1 |
| Palmatine | CSF1R |
| Palmatine | ESR1 |
| Palmatine | ESR2 |
| Palmatine | LRRK2 |
| Palmatine | MMP9 |
| Palmatine | MTOR |
| Palmatine | NOS2 |
| Palmatine | NOS3 |
| Palmatine | NTRK1 |
| Palmatine | PDGFRB |
| Palmatine | PIK3CB |
| Palmatine | PIK3CD |
| Palmatine | PIK3CG |
| Palmatine | PTGS2 |
| Palmatine | SCN5A |
| Palmatine | SIGMAR1 |
| Worenine | ACHE |
| Worenine | BCHE |
| Worenine | CHRM1 |
| Worenine | ESR1 |
| Worenine | NOS2 |
| Worenine | PTGS2 |
| Worenine | SIGMAR1 |
